# Assessing the Protective Role of Cheese Consumption Against Type 2 Diabetes and Its Complications: A Mendelian Randomization Study

**DOI:** 10.1155/ije/8880270

**Published:** 2025-07-18

**Authors:** Shuwei Weng, Xin Guo, Chen Ding, Die Hu, Daoquan Peng

**Affiliations:** ^1^Department of Cardiovascular Medicine, The Second Xiangya Hospital, Central South University, Changsha, Hunan, China; ^2^Research Institute of Blood Lipid and Atherosclerosis, Central South University, Changsha, Hunan, China; ^3^Department of Cardiology, The First Affiliated Hospital of Fujian Medical University, Fuzhou, Fujian, China

**Keywords:** cheese consumption, diabetic complications, dietary intervention, Mendelian randomization, type 2 diabetes mellitus

## Abstract

**Background:** Type 2 diabetes mellitus (T2DM) is a major global health issue, with significant complications impacting patients' quality of life, including neuropathy, ophthalmic issues, nephropathy, and peripheral vascular complications. Although dietary factors influence T2DM risk, the specific impact of cheese consumption remains unclear. This study uses a two-sample Mendelian randomization (MR) approach to investigate the causal relationship between cheese intake and T2DM, along with specific complications, including ophthalmic and peripheral vascular issues.

**Methods:** Using summary-level data from large-scale genome-wide association studies, we applied a two-sample MR approach. Genetic variants associated with cheese consumption were selected as instrumental variables, following criteria for genome-wide significance, linkage disequilibrium checks, and exclusion of pleiotropic effects. Robustness was assessed through various MR methods, including inverse variance weighted (IVW) and MR-Egger.

**Results:** The MR analysis found that increased cheese consumption was significantly associated with a reduced risk of T2DM (OR = 0.639, 95% CI: 0.482–0.847, and *p* value = 0.002) and its ophthalmic complications (OR = 0.386, 95% CI: 0.196–0.759, and *p* value = 0.015). No significant associations were found with other complications, including neuropathy, nephropathy, and peripheral vascular complications. Sensitivity analyses confirmed minimal heterogeneity and pleiotropy, supporting the reliability of these findings.

**Conclusion:** This study suggests a protective role of cheese intake in reducing T2DM risk and its ophthalmic complications, potentially informing dietary recommendations for T2DM management.

## 1. Introduction

Type 2 diabetes mellitus (T2DM) is a chronic metabolic disorder characterized by insulin resistance and hyperglycemia [1]. According to epidemiological statistics, approximately 1 in 11 adults worldwide has diabetes, with 90% of cases being T2DM [2]. T2DM is a major contributor to the global burden of noncommunicable diseases and is closely associated with various systemic complications, including cardiovascular, renal, and neurological disorders [3]. Among these complications, diabetic neuropathy, ophthalmic complications, peripheral circulatory issues, and kidney complications represent some of the most common and severe outcomes of poorly managed T2DM, significantly impacting patients' quality of life [4].

Recent research has increasingly focused on dietary factors that could modulate the risk or progression of T2DM and its complications [5–7]. Dairy consumption, particularly cheese, has been a topic of interest due to its complex nutritional profile. While cheese is rich in saturated fats, it also provides beneficial nutrients such as calcium, protein, and bioactive compounds that may positively influence metabolic health [8–10]. In our previous two-sample Mendelian randomization (MR) study, we identified a potential causal relationship between cheese consumption and a lower risk of nonalcoholic fatty liver disease [11]. Several observational studies have suggested that dairy intake, including cheese, may lower the risk of T2DM, possibly due to its role in weight management and improving insulin sensitivity [12]. However, the causal relationship between cheese consumption and T2DM, particularly with its neurological, renal, and circulatory complications, remains uncertain due to potential confounding factors.

To address this gap, MR has emerged as a robust method for inferring causality between exposures (like dietary habits) and health outcomes by using genetic variants as instrumental variables [13, 14]. This technique minimizes the bias caused by confounding variables, thus providing more reliable causal inferences. While MR studies have explored the role of dairy in various health outcomes, limited research has focused on cheese consumption's specific effects on T2DM and its complications.

In this MR study, we aim to investigate the causal relationship between cheese intake and the risk of T2DM and its related complications, including diabetic neuropathy, ophthalmic complications, peripheral circulatory disorders, and nephropathy. By leveraging genetic variants as instrumental variables, this study provides new insights into the dietary recommendations for preventing T2DM complications and improving metabolic health outcomes.

## 2. Materials and Methods

### 2.1. MR

**Figure 1:**
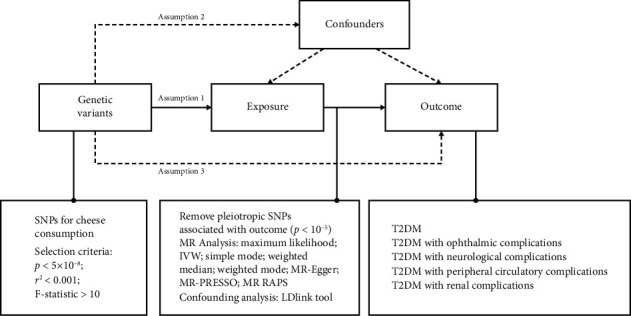
Plot of three key assumptions.

This study utilized a two-sample MR design, drawing on summary-level data to explore the causal relationship between cheese intake and T2DM complications. The MR approach (Figure 1) is grounded in three key assumptions about genetic variants: Assumption 1 is that the variants must be strongly associated with the exposure of interest (relevance assumption); Assumption 2 is that they should be independent of any confounding variables that could affect the exposure–outcome relationship (independence assumption); and Assumption 3 is that their effect on the outcome should occur only through the exposure, with no alternative pathways involved (exclusion restriction assumption) [15].

### 2.2. Selection of Exposure and Outcome Variables

We selected exposure and outcome data from large-scale genome-wide association studies (GWAS), compiling essential characteristics such as GWAS-ID, PMID, first author, year, population demographics, sample size, variant count, and consortium information (Table 1). This MR analysis was focused on European populations to ensure consistency and avoid cohort overlap. The exposure of interest was cheese intake, while the outcomes included T2DM complications such as ophthalmic, neurological, peripheral circulatory, and renal complications.

### 2.3. Selection and Validation of Single Nucleotide Polymorphisms (SNPs)

SNPs associated with cheese intake were identified using a genome-wide significance threshold (*p* value < 5 × 10^−8^). We further ensured that the selected SNPs were independent of each other by conducting linkage disequilibrium (LD) checks, removing SNPs with an LD *r*^2^ > 0.001. To maintain robust instrument quality, only SNPs with an F-statistic greater than 10 were included (Supporting Table 1). In addition, to minimize the potential influence of confounders, we identified SNPs associated with traits that could bias the results using the LDlink tool (https://ldlink.nih.gov/?tab=ldtrait). The detailed LDlink results for the instrumental variables are provided in Supporting Table 2. Potential confounding associations identified in previous GWAS studies are listed in Supporting Table 3, and the final inclusion or exclusion of SNPs after quality control is summarized in Supporting Table 1. After excluding SNPs associated with GWAS traits listed in Supporting Table 3, we further examined the remaining candidate SNPs using the LDtrait module and found no significant associations with lipid metabolism or lactose metabolism traits.

### 2.4. MR Analysis

Multiple methods were employed in the MR analysis to test the causal relationship between cheese intake and T2DM complications. The multiplicative random effects inverse variance weighted (IVW) method was chosen as the primary analysis due to its high precision and accuracy in estimating causal effects. Complementary MR methods, including maximum likelihood, IVW (fixed effects), simple mode, weighted median, weighted mode, MR-Egger, MR-PRESSO, and MR RAPS, were used to further support the robustness of the findings and assess potential biases such as pleiotropy.

### 2.5. Sensitivity Analysis and Pleiotropy Analysis

Sensitivity analyses were conducted to evaluate the robustness of the MR results. We used MR-Egger and IVW methods to perform Cochran's *Q* test to assess heterogeneity among the individual SNPs. The MR-Egger intercept test was also used to detect horizontal pleiotropy, where a significant intercept indicates pleiotropic effects. In addition, the MR-PRESSO method was employed to further investigate and correct for pleiotropy. To identify SNPs with a disproportionate influence on the MR results, a leave-one-out analysis was conducted. Scatter plots and funnel plots were generated to visualize the associations between each SNP and the outcome, as well as to detect heterogeneity and pleiotropy in the MR analysis.

### 2.6. Statistical Analysis

All statistical analyses were performed using R software (Version 4.1.2). The MR analyses were conducted using the “TwoSampleMR” package (Version 0.5.11), including Steiger directionality tests performed using the Steiger filtering function, with the MR-PRESSO method implemented through the “MRPRESSO” package (Version 1.0) and the robust adjusted profile score (RAPS) method applied using the “mr.raps” package (Version 0.2). A *p* value of less than 0.05 was considered statistically significant across all primary analyses, as well as in the pleiotropy and sensitivity analyses.

## 3. Results

### 3.1. Assessment of MR Analysis Results

**Figure 2:**
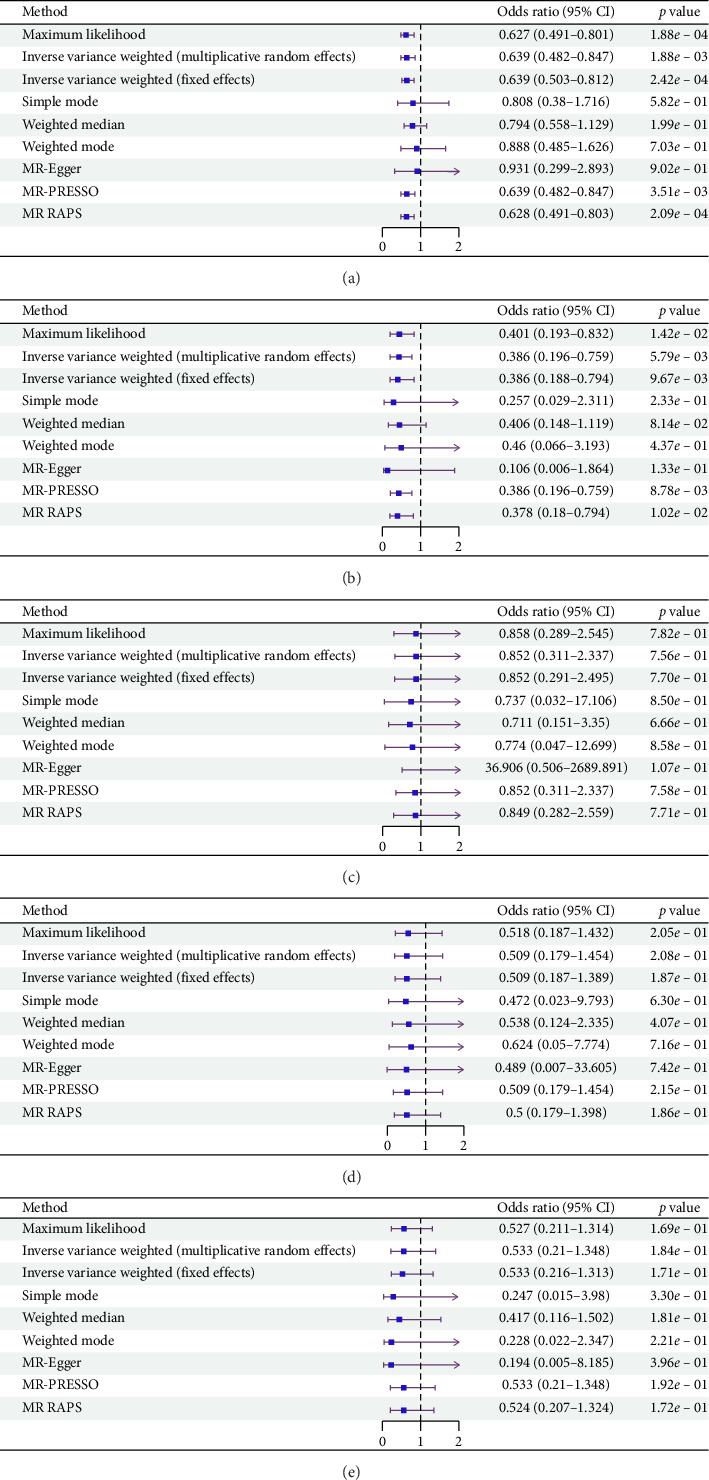
The forest plot of the causal effect of cheese intake on different outcome variables. (a) T2DM; (b) T2DM with ophthalmic complications; (c) T2DM with neurological complications; (d) T2DM with peripheral circulatory complications; and (e) T2DM with renal complications.

**Figure 3:**
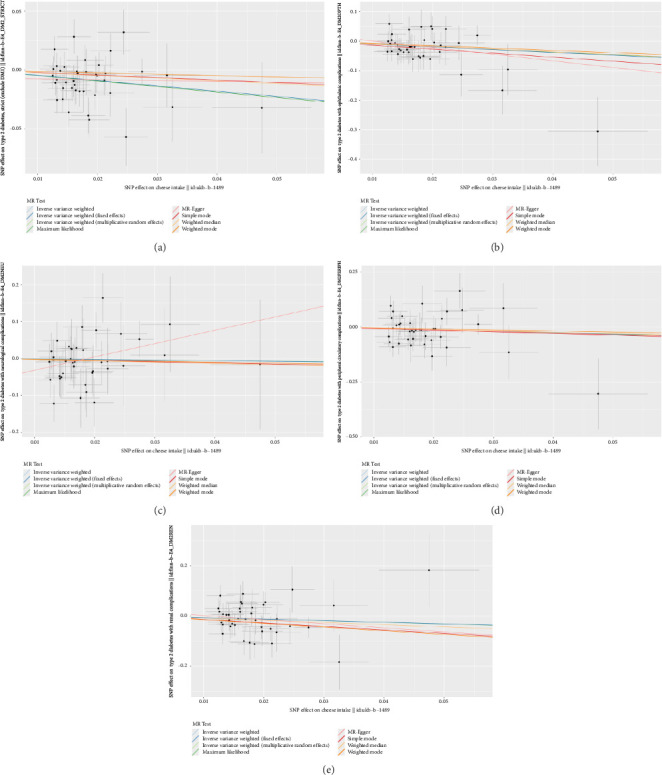
Scatter plot of MR analysis using different MR methods. (a) T2DM; (b) T2DM with ophthalmic complications; (c) T2DM with neurological complications; (d) T2DM with peripheral circulatory complications; and (e) T2DM with renal complications.

According to the results of the multiplicative random effects IVW method, genetically predicted cheese consumption, increased by one standard deviation, was negatively associated with the risk of T2DM (OR = 0.639, 95% CI: 0.482–0.847, *p* value = 0.002). This association was also observed using the maximum likelihood, fixed-effects IVW, MR-PRESSO, and MR RAPS methods. In addition, cheese intake was significantly negatively associated with ophthalmic complications of T2DM (OR = 0.386, 95% CI: 0.196–0.759, and *p* value = 0.015). However, cheese intake did not appear to be significantly associated with T2DM complications such as neurological complications, peripheral circulatory complications, or renal complications (OR = 0.852, 95% CI: 0.311–2.337, and *p* value = 0.756; OR = 0.509, 95% CI: 0.179–1.454, and *p* value = 0.208; and OR = 0.533, 95% CI: 0.210–1.348, and *p* value = 0.184, respectively) (Figure 2, Supporting Figure 2, and Supporting Table 4). The scatter plot (Figure 3) clearly illustrates the negative correlation between cheese intake and both T2DM and its ophthalmic complications. It is worth noting that methods other than the multiplicative random effects IVW have limitations regarding statistical assumptions and precision. Therefore, a lack of statistical significance in certain methods does not imply an absence of association between exposure and outcome variables. The observed significant associations serve only as indicative evidence. Our MR analysis is primarily based on the results of the multiplicative random effects IVW method, with other methods providing supportive evidence. Steiger directionality tests further confirmed that all 40 genetic variants used as instrumental variables explained more variance in cheese intake than in the outcomes. This result supports the assumed causal direction from cheese intake to T2DM and its complications and strengthens the validity of the MR framework employed in this study (Table 2).

### 3.2. Sensitivity Analysis and Pleiotropy Assessment

In this study, we conducted a heterogeneity analysis of the relationship between cheese intake and T2DM, as well as its complications. While the Cochran's *Q* test under the MR-Egger method yielded a *p* value of 0.046 for the association between cheese intake and T2DM, indicating marginal heterogeneity, this finding should be interpreted cautiously. The MR-Egger method is known to be more sensitive to minor heterogeneity and may produce inflated variance estimates, especially when the number of instrumental variables is modest. Importantly, the IVW method, which served as the primary analytic model in this study, did not indicate significant heterogeneity (*p* ≥ 0.05), suggesting that the causal estimates are relatively stable. Furthermore, sensitivity analyses including MR-PRESSO, leave-one-out tests, and funnel plot assessments did not reveal substantial pleiotropy or influential SNPs, reinforcing the robustness of the observed associations. Similarly, for the relationship between cheese intake and other T2DM-related complications, the *p* values of Cochran's *Q* test were greater than 0.05 using both the IVW and MR-Egger methods, indicating no significant heterogeneity in these results. Overall, these findings support the robustness of the model assumptions, suggesting relatively consistent effects of instrumental variables in the causal inference between cheese intake and T2DM, as well as its complications, with minimal or negligible horizontal pleiotropy (Table 2). The symmetric distribution of points in the funnel plot further supports the conclusion of no significant heterogeneity (Supporting Figure 3). Leave-one-out analysis indicated that the removal of any single SNP did not affect the significance of the results, indicating the absence of potential pleiotropy (Supporting Figure 1). However, the MR-Egger intercept test did not provide evidence of pleiotropy in the relationship between cheese intake and T2DM or its complications. This finding is consistent with the overall test results of MR-PRESSO (Table 2).

## 4. Discussion

In this two-sample MR study, we found a negative association between cheese intake and the occurrence of type 2 diabetes and its ophthalmic complications, based on large-scale GWAS summary statistics and results calculated using the IVW method with a random effects model. A recent MR study by Zhong et al. also investigated the causal relationship between cheese intake and T2DM, reporting a similar inverse association. The consistency of our findings with those of Zhong et al. reinforces the robustness of this causal relationship [16]. However, our study extends the existing evidence by further exploring the potential impact of cheese consumption on T2DM-related complications, providing additional insights into its metabolic benefits.

Cheese is a versatile dairy product that is typically an important source of protein and fat in the Mediterranean diet. Traditionally, all saturated fatty acids have been considered potentially harmful to health [17–19], but recent literature suggests that dairy-derived fatty acids may play a protective role in diabetes prevention [20]. Current research on cheese and T2DM is predominantly observational. For example, the prospective cohort EPIC-InterAct study based on a European population found that total dairy intake was not associated with diabetes, but among dairy subtypes, cheese intake tended to be negatively associated with diabetes [21]. A cross-sectional study from Brazil indicated that higher intake of dairy products, especially full-fat dairy, was associated with lower blood glucose levels [22]. This association was more pronounced among overweight adolescents, while low-fat dairy products were associated with a higher prevalence of prediabetes and T2DM. A meta-analysis of 25 studies including a total of 718,691 subjects showed a negative association between cheese consumption and the risk of T2DM; however, a dose-response meta-analysis did not support a negative association between cheese consumption and T2DM risk [23]. A longitudinal study from Australia involving 8122 participants found that higher cheese consumption was associated with a lower risk of diabetic retinopathy progression. The hazard ratios (HR) for the highest quartile compared to the lowest quartile were 0.58 (95% CI: 0.41–0.83; *p* value = 0.007) and 0.64 (95% CI: 0.46–0.89; *p* value = 0.04), respectively [24].

Although our analysis using genetic variants as instrumental variables cannot directly explain this mechanism, existing literature provides several possible biological explanations. First, phenolic compounds in cheese, especially in cheeses enhanced with herbs, fruits, or fungi, have significant antioxidant activity. These compounds can inhibit key enzymes related to T2DM, such as α-glucosidase and α-amylase, potentially reducing insulin resistance [25]. In addition, cheese acts as a carrier for probiotics and prebiotics, which can modulate the gut microbiota, improve the gut microenvironment, reduce oxidative stress and inflammation, and enhance insulin sensitivity—a metabolic regulatory function with potential benefits in T2DM management [26]. Recent MR studies using a two-step mediation approach have revealed that certain gut microbiota taxa, notably members of Ruminococcaceae UCG005 and UCG010, causally influence plasma metabolite concentrations—including octadecadienedioate (C18:2-DC)—and these metabolite changes in turn are significantly associated with lower risk of type 2 diabetes, thereby elucidating an intermediate gut-microbiota-to-metabolite-to-diabetes causal pathway [27]. Further research has shown that dairy fat in cheese can activate the intracellular AMPK signaling pathway, reducing cellular energy overload and mitigating inflammation caused by lipid accumulation, thus protecting β-cells and preventing insulin resistance [28]. Moreover, the antioxidant components in cheese may reduce mitochondrial reactive oxygen species (ROS) production, inhibit cell stress pathways such as the JNK pathway, reduce insulin signaling inhibition, and improve insulin sensitivity [29]. Diabetic retinopathy is the primary manifestation of diabetic ophthalmic complications, and its occurrence is closely associated with the involvement of the inflammatory factor TNF-α. Studies have shown that TNF-α can promote abnormal retinal angiogenesis and exacerbate disease progression by activating the JNK signaling pathway and upregulating RUNX1 expression in endothelial cells. Moreover, the synergistic effects of TNF-α and high glucose concentrations amplify this process through the JNK-AP-1-RUNX1 feedback loop. VEGF regulation can partially inhibit the pathogenic effects of TNF-α, suggesting that the TNF-α signaling pathway is a critical therapeutic target for diabetic retinopathy [30]. A randomized crossover trial in patients with metabolic syndrome demonstrated that the intake of dairy products, including cheese, has significant anti-inflammatory effects, markedly reducing plasma TNF-α levels [31]. Building on evidence from observational studies and biological mechanisms suggesting a protective role of cheese in diabetes and its complications, our MR analysis provides stronger causal inferences by utilizing genetic instruments. These findings collectively highlight that cheese intake is negatively associated with T2DM and its ophthalmic complications, with potential benefits mediated through antioxidant and anti-inflammatory mechanisms, thereby offering valuable insights for the prevention and management of T2DM and its complications [32].

Notably, our MR analysis did not detect significant associations between cheese intake and diabetic neuropathy, nephropathy, or peripheral circulatory complications. This may reflect both distinct pathophysiological mechanisms and limited statistical power. Diabetic neuropathy is driven by axonal degeneration, segmental demyelination, oxidative stress, neuroinflammation, and microvascular dysfunction in peripheral nerves [33]. In contrast, nephropathy involves glomerular injury, renal inflammation, fibrosis, and perturbations of the gut–kidney axis mediated by microbial metabolites and uremic toxins [34, 35]. Peripheral circulatory complications, particularly peripheral artery disease in T2DM, primarily result from endothelial dysfunction and accelerated atherosclerosis. Persistent hyperglycemia promotes oxidative stress and inflammation via activation of NADPH oxidase and NF-κB, while advanced glycation end products deposit in vessel walls, reducing nitric oxide bioavailability and leading to smooth muscle proliferation and plaque formation [36]. Although cheese contains bioactive components—calcium, probiotic bacteria, and antioxidant peptides—that have demonstrated benefits for glycemic control, oxidative stress reduction, and microvascular health, these compounds may not strongly influence the specific molecular pathways underlying nerve damage, renal fibrosis, or macrovascular plaque formation [37, 38]. Moreover, the sample sizes for these complications are substantially smaller than those for overall T2DM or ophthalmic complications (Table 1), which likely reduces the statistical power to detect more modest causal effects. Together, these factors suggest that the lack of significant findings may represent both mechanistic specificity and insufficient power in the available datasets. Future studies utilizing larger, complication-specific GWAS datasets and mechanistic approaches—such as gene–metabolite mediation MR—are warranted to determine whether protective effects of cheese extend to these complications.

This study has some limitations. First, the data used in this study are entirely from European populations. Differences in allele frequencies, LD structures, and gene–environment interactions across ethnic groups may result in population-specific genetic effects, thereby limiting the generalizability of our findings to other populations such as Asians or Africans. Future MR studies incorporating multiethnic cohorts are needed to validate and expand upon these results. Second, although MR can reduce the impact of confounding factors at the genetic level and thus enhance the reliability of causal inference, it cannot completely rule out the potential interactions between genetic variants and environmental factors that may influence the results. Lastly, while MR provides strong evidence of causality, it remains challenging to uncover the underlying biological mechanisms of the association between cheese intake and T2DM and its complications.

## 5. Conclusion

This MR study suggests a protective association between cheese intake and reduced risk of type 2 diabetes and its ophthalmic complications. These findings highlight the potential role of cheese as part of a balanced diet to support metabolic health and may inform dietary recommendations for type 2 diabetes management. Further research into the biological mechanisms underlying this relationship could provide additional insights into dietary approaches for preventing T2DM complications.

## Figures and Tables

**Table 1:** Characteristics of data of cheese intake and T2DM and its complications.

	Exposures/outcomes	GWAS-ID	Details
Exposure	Cheese intake	ukb-b-1489	Year: 2018
			Author: Ben Elsworth
			Population: European
			Sex: males and females
			Sample size: 451,486
			Number of SNPs: 8,851,867
			Consrtium: MRC-IEU

Outcome	T2DM	finn-b-E4_DM2_STRICT	Year: 2021
			Author: NA
			Population: European
			Sex: males and females
			Sample size: case 29,166; control 183,185
			Number of SNPs: 16,380,434
			Consrtium: NA
	T2DM with ophthalmic complications	finn-b-E4_DM2OPTH	Year: 2021
			Author: NA
			Population: European
			Sex: males and females
			Sample size: case 2119; control 183,185
			Number of SNPs: 16,380,340
			Consrtium: NA
	T2DM with neurological complications	finn-b-E4_DM2NEU	Year: 2021
			Author: NA
			Population: European
			Sex: males and females
			Sample size: case 911; control 183,185
			Number of SNPs: 16,380,335
			Consrtium: NA
	T2DM with peripheral circulatory complications	finn-b-E4_DM2PERIPH	Year: 2021
			Author: NA
			Population: European
			Sex: males and females
			Sample size: case 1049; control 183,185
			Number of SNPs: 16,380,336
			Consrtium: NA
	T2DM with renal complications	finn-b-E4_DM2REN	Year: 2021
			Author: NA
			Population: European
			Sex: males and females
			Sample size: case 1296; control 183,185
			Number of SNPs: 16,380,337
			Consrtium: NA

**Table 2:** Pleiotropy, heterogeneity, and directionality tests.

	Test	Method	Results
T2DM	Pleiotropy	MR-PRESSO	RSSobs (Global test): 57.22
			*p* value (global test): 6.00 × 10^−2^
		MR-Egger intercept test	Estimated value of the intercept: −6.79 × 10^−3^
			Standard error: 1.01 × 10^−2^
			*p* value: 5.06 × 10^−1^
	Heterogeneity	MR-Egger	Cochran's *Q*: 53.77
			Degree of freedom: 38
			^∗^ *p* value: 4.65 × 10^−2^
		IVW	Cochran's *Q*: 54.41
			Degree of freedom: 39
			*p* value: 5.16 × 10^−2^
	Directionality	Steiger test	Number of SNPs included: 40

T2DM with ophthalmic complications	Pleiotropy	MR-PRESSO	RSSobs (global test): 36.23
			*p* value (global test): 6.97 × 10^−1^
		MR-Egger intercept test	Estimated value of the intercept: 2.34 × 10^−2^
			Standard error: 2.56 × 10^−2^
			*p* value: 3.66 × 10^−1^
	Heterogeneity	MR-Egger	Cochran's *Q*: 33.46
			Degree of freedom: 38
			*p* value: 6.79 × 10^−1^
		IVW	Cochran's *Q*: 34.30
			Degree of freedom: 39
			*p* value: 6.84 × 10^−1^
	Directionality	Steiger test	Number of SNPs included: 40

T2DM with neurological complications	Pleiotropy	MR-PRESSO	RSSobs (global test): 36.50
			*p* value (global test): 6.82 × 10^−1^
		MR-Egger intercept test	Estimated value of the intercept: −6.80 × 10^−2^
			Standard error: 3.82 × 10^−2^
			*p* value: 8.33 × 10^−2^
	Heterogeneity	MR-Egger	Cochran's *Q*: 31.22
			Degree of freedom: 38
			*p* value: 7.74 × 10^−2^
		IVW	Cochran's *Q*: 34.38
			Degree of freedom: 39
			*p* value: 6.80 × 10^−1^
	Directionality	Steiger test	Number of SNPs included: 40

T2DM with peripheral circulatory complications	Pleiotropy	MR-PRESSO	RSSobs (Global test): 44.67
			p value (global test): 3.30 × 10^−1^
		MR-Egger intercept test	Estimated value of the intercept: 7.49 × 10^−4^
			Standard error: 3.77 × 10^−2^
			*p* value: 9.84 × 10^−1^
	Heterogeneity	MR-Egger	Cochran's *Q*: 42.65
			Degree of freedom: 38
			*p* value: 2.78 × 10^−1^
		IVW	Cochran's *Q*: 42.65
			Degree of freedom: 39
			*p* value: 3.17 × 10^−1^
	Directionality	Steiger test	Number of SNPs included: 40

T2DM with renal complications	Pleiotropy	MR-PRESSO	RSSobs (Global test): 43.48
			*p* value (global test): 0.37
		MR-Egger intercept test	Estimated value of the intercept: 1.82 × 10^−2^
			Standard error: 3.34 × 10^−2^
			*p* value: 5.88 × 10^−1^
	Heterogeneity	MR-Egger	Cochran's *Q*: 41.04
			Degree of freedom: 38
			*p* value: 3.39 × 10^−1^
		IVW	Cochran's *Q*: 41.37
			Degree of freedom: 39
			*p* value: 3.68 × 10^−1^
	Directionality	Steiger test	Number of SNPs included: 40

Abbreviation: RSSobs, observed residual sum of squares.

^∗^Indicates statistical significance at *p* < 0.05.

## Data Availability

The data used in this study are publicly available from the IEU OpenGWAS database (https://gwas.mrcieu.ac.uk/). All datasets utilized for the MR analyses can be accessed through this platform. Further details regarding the datasets are provided in the manuscript and are available upon request from the corresponding author.

## Supporting Information

(1) Supporting Figures

Supporting Figure 1: Leave-one-out plots. (a) T2DM; (b) T2DM with ophthalmic complications; (c) T2DM with neurological complications; (d) T2DM with peripheral circulatory complications; and (e) T2DM with renal complications.

Supporting Figure 2: Visual SNPs plots. (a) T2DM; (b) T2DM with ophthalmic complications; (c) T2DM with neurological complications; (d) T2DM with peripheral circulatory complications; and (e) T2DM with renal complications.

Supporting Figure 3: Funnel plots. (a) T2DM; (b) T2DM with ophthalmic complications; (c) T2DM with neurological complications; (d) T2DM with peripheral circulatory complications; and (e) T2DM with renal complications.

(2) Supporting Tables

Supporting Table 1: Characteristics of instrumental variables.

Supporting Table 2: The LDlink results of the instrumental variables used in this study.

Supporting Table 3: Potential confounding associations of instrumental variable SNPs.

Supporting Table 4: Feature variable selection table.
