# Supplementary material for: Assessing the Protective Role of Cheese Consumption Against Type 2 Diabetes and Its Complications: A Mendelian Randomization Study
Source: Int J Endocrinol. 2025 Jul 18;2025:8880270. doi: 10.1155/ije/8880270 (PMC12297135; doi:10.1155/ije/8880270)
Supplement: Supporting Information — Additional supporting information can be found online in the Supporting Information section. [file 8880270.f1.docx]

Supporting Information

# Supporting Figures


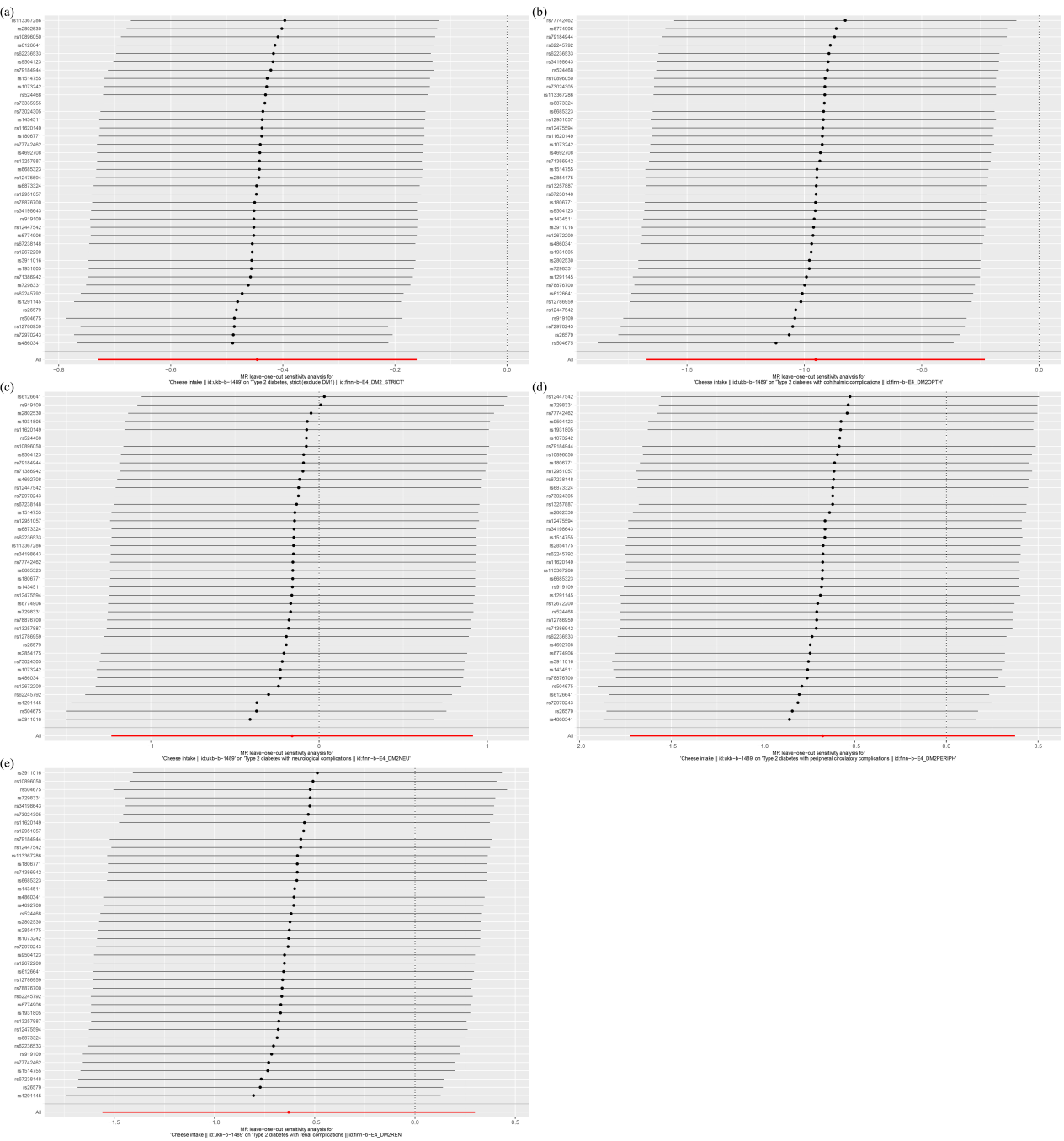


**Supporting Figure 1.** Leave-one-out plots.

(a) T2DM; (b) T2DM with ophthalmic complications; (c) T2DM with neurological complications; (d) T2DM with peripheral circulatory complications; (e) T2DM with renal complications


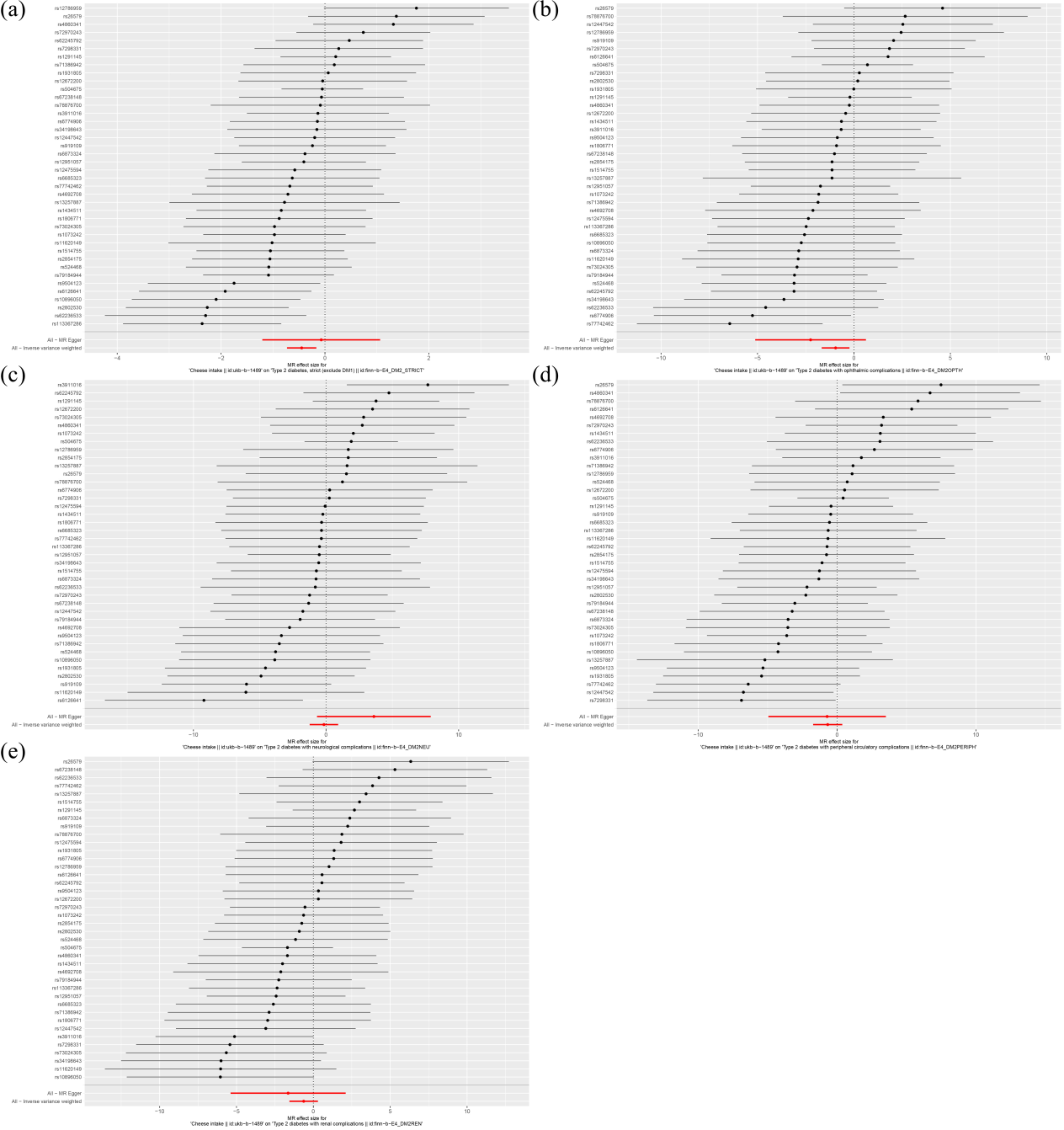


**Supporting Figure 2.** Visual SNPs plots.

(a) T2DM; (b) T2DM with ophthalmic complications; (c) T2DM with neurological complications; (d) T2DM with peripheral circulatory complications; (e) T2DM with renal complications


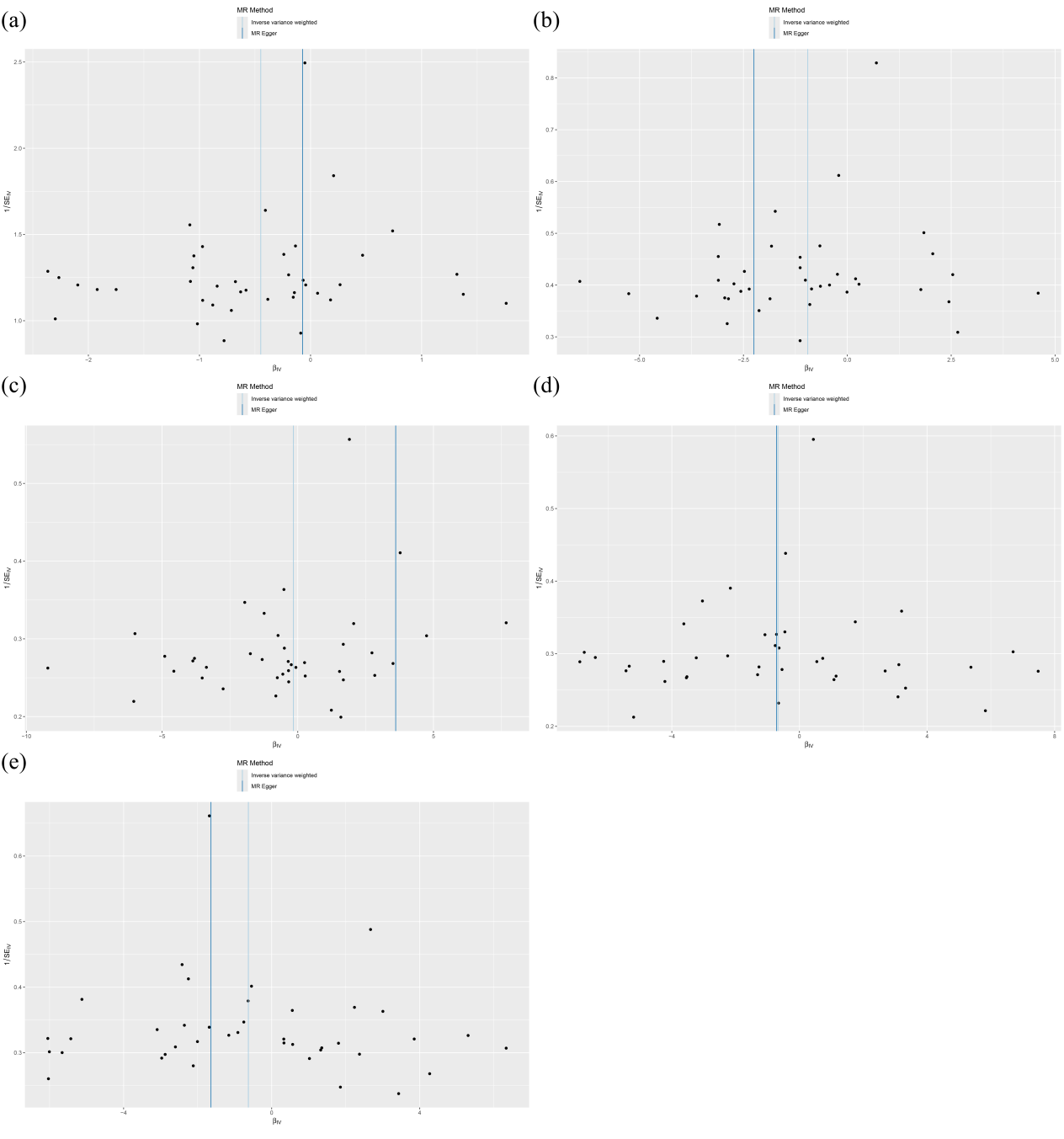


**Supporting Figure 3.**  Funnel plots.

(a) T2DM; (b) T2DM with ophthalmic complications; (c) T2DM with neurological complications; (d) T2DM with peripheral circulatory complications; (e) T2DM with renal complications

# Supporting Tables

（Supporting Table 1, 2 and 3 are too large to be displayed completely in this document. For details, please refer to the editable CSV files uploaded.）

**Supporting Table 1.** Characteristics of instrumental variables.

**Supporting Table 2.** The LDlink results of the instrumental variables used in this study

**Supporting Table 3.** Potential Confounding Associations of Instrumental Variable SNPs

**Supporting Table 4.** Feature Variable Selection Table
